# Refractory systemic juvenile idiopathic arthritis complicated by coxarthrosis

**DOI:** 10.1186/1546-0096-12-S1-P232

**Published:** 2014-09-17

**Authors:** Antonio Mellos, Angela Mauro, Carmela Granato, Maria Francesca Gicchino, Laura Perrone, Alma Nunzia Olivieri

**Affiliations:** 1 Department of the Women, Child, General and Specialized Surgery, Second University of Naples, Italy , Naples, Italy

## Introduction

S-JIA has the worst long-term prognosis compared to other types of JIA. Corticosteroids, methotrexate and anti - tumor necrosis factor (TNF) are the most commonly used drugs for it. Recently, newer biologic agents targeting IL-1 and IL-6 have proven their effectiveness in treating s-JIA and in minimizing corticosteroid exposure.

## Objectives

To describe the challenging management of a case of refractory s-JIA.

## Methods

Laboratory tests and diagnostic imaging were performed taking into account the history and the pathological findings highlighted at the physical examination of a 14 year old girl febrile (38 ° C) since about a month, especially in the evening. She appeared pale, suffering from right arthritis of elbow, knee, hip and left arthritis of the ankle and at the third finger of the hand. Diagnosis of s-JIA was made according with ILAR classification criteria (second revision, Edmonton 2001).

## Results

Blood tests found : inflammatory anemia (Hb 7.5 g/dl, ESR 128 mm/1h, CRP 32 mg/dl, ferritin 485 ng/dl; fibrinogen 634 mg/dl); normal WBC count; absence of thrombocytosis; normal PT and aPTT, hypoalbuminemia (3,1 gr/dl) and an increase of FDP (> 20 μg/l) and D-dimer (8 μg/dl). Blood culture and urine test were sterile, ANA and ENA were absent. Eye examination excluded uveitis. Diagnostic imaging didn’t found pathological signs. Bone marrow aspiration excluded a primary or metastatic neoplastic disease and hemophagocytosis. Suspecting a s-JIA, we decided to administer intravenous methylprednisolone (1 g) for two days, followed by the oral administration of prednisone (1.3 mg/kg/day), resulting in a marked improvement of the clinical symptoms and blood parameters in seven days. We associated methotrexate (MTX, 10 mg/m²/week) and naproxen with steroid therapy, we tried several times to climb the steroid dose and reduce it to less than 0.3 mg/kg, but without success. In march 2011, we decided to add anakinra (100 mg/day subcutaneously) to her therapeutic regimen because of the corticodependence and the poor control of the disease (acute arthrosynovitis of left knee; functional limitation of both hips; Hb 10,5 gr/dl; ESR 35 mm/1h; CRP 3,3 mg/dl). After an initial clinical response (absence of acute arthrosynovitis; Hb 11,2 gr/dl; ESR 11 mm/1h; CRP 0,33 mg/dl), Anakinra has been suspended in july 2011 following the appearance of local reactions at the injection site compromising its administration. It has been replaced with etanercept (0.4 mg/kg, administered twice per week by subcutaneous injection). In april 2012, because of secondary unilateral coxarthrosis, the patient has undergone a cementless total right hip replacement. The disease, initially responsive to etanercept (Hb 12,2 gr/dl; ESR 18mm/1h; CRP 0,12 mg/dl ), also became refractory to this biologic drug ( arthrosynovitis acute of the right knee and left ankle; functional limitation of both elbows; Hb 10,8mg/dl; ESR 95mm/1h; CRP 11 mg/dl). Then, in october 2012 the anti-TNF therapy was discontinued and replaced with canakinumab (150 mg/day subcutaneously), initially effective, but suspended in may 2014 due to the refractory disease. Currently, the patient has acute arthrosynovitis of both knees even though they have already been infiltrated with triamcinolone acetonide, a functional limitation of the left hip and an inflammatory anemia (Hb 10.3 g / dl; VES 75mm/1h; CRP 8 mg/dl, SAA 99 mg/L). Her current treatment is prednisone (0.4 mg/kg/day) associated with indomethacin and MTX.

## Conclusion

Considering the efficacy of anti-IL-6 blockade described in s-JIA, we're going to start the intravenous infusion of Tocilizumab (8 mg/kg over 60 minutes).

## Disclosure of interest

None declared.

